# The Anatomy, Development, and Evolution of the Atrioventricular Conduction Axis

**DOI:** 10.3390/jcdd5030044

**Published:** 2018-08-22

**Authors:** Robert H. Anderson, Shumpei Mori, Diane E. Spicer, Damian Sanchez-Quintana, Bjarke Jensen

**Affiliations:** 1 Institute of Genetic Medicine, Newcastle University, Newcastle upon Tyne NE1 4EP, UK; sejjran@ucl.ac.uk; 2 Division of Cardiovascular Medicine, Department of Internal Medicine, Kobe University Graduate School of Medicine, 7-5-1 Kusunoki-cho, Chuo-ku, Kobe 650-0017, Hyogo, Japan; shumpei_8@hotmail.com; 3 Department of Pediatric Cardiology, University of Florida, Gainesville, FL 32610, USA; spicerpath@hotmail.com; 4 Department of Anatomy and Cell Biology, Faculty of Medicine, University of Extremadura, 06006 Badajoz, Spain; damians@unex.es; 5 University of Amsterdam, Amsterdam UMC, Department of Medical Biology, Amsterdam Cardiovascular Sciences, Meibergdreef 15, 1105AZ Amsterdam, The Netherlands

**Keywords:** atrioventricular node, bundle of His, ventricular bundle branches, retroaortic node, dead-end tract

## Abstract

It is now well over 100 years since Sunao Tawara clarified the location of the axis of the specialised myocardium responsible for producing coordinated ventricular activation. Prior to that stellar publication, controversies had raged as to how many bundles crossed the place of the atrioventricular insulation as found in mammalian hearts, as well as the very existence of the bundle initially described by Wilhelm His Junior. It is, perhaps surprising that controversies continue, despite the multiple investigations that have taken place since the publication of Tawara’s monograph. For example, we are still unsure as to the precise substrates for the so-called slow and fast pathways into the atrioventricular node. Much has been done, nonetheless, to characterise the molecular make-up of the specialised pathways, and to clarify their mechanisms of development. Of this work itself, a significant part has emanated from the laboratory coordinated for a quarter of a century by Antoon FM Moorman. In this review, which joins the others in recognising the value of his contributions and collaborations, we review our current understanding of the anatomy, development, and evolution of the atrioventricular conduction axis.

## 1. Introduction

**Figure 1:**
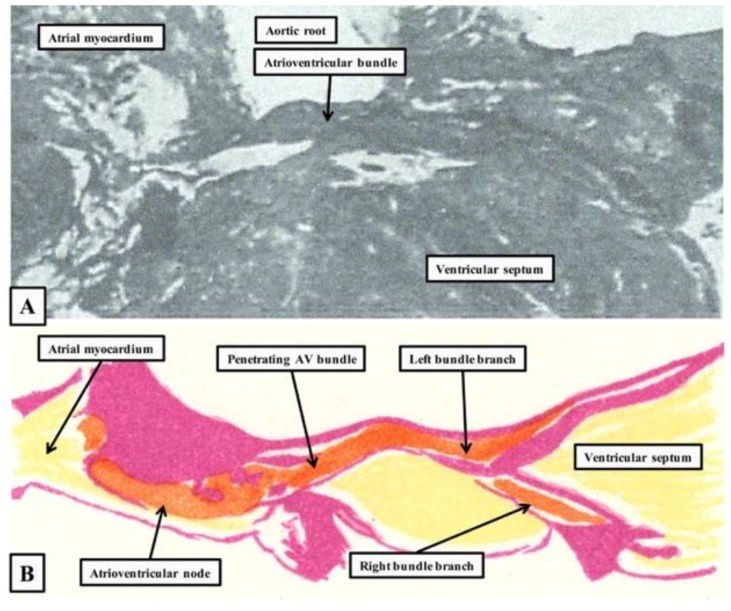
The figure is prepared by scanning the figures from the original publication of His [6] (**A**) and the monograph of Tawara [1] (**B**). His’s figure is shown as seen from the right side, with the heart in “Valentine” orientation. Tawara’s figure is re-orientated to show the arrangement as seen in attitudinally appropriate fashion, with the endocardial surface of the right-sided chambers to the bottom of the picture. His’ image shows the penetrating portion of the atrioventricular (AV) conduction axis, whereas the figure of Tawara shows its overall extent.

The identification of the atrioventricular conduction axis by Sunao Tawara, in 1906 [1] ushered in the correct understanding of cardiac conduction [2,3]. As was stated by Keith, stimulated by the findings, and who together with Flack was shortly after to identify the sinus node [4], “with the discovery of the conduction system of Tawara, heart research entered a new epoch” [5]. Prior to Tawara’s publication, His had already described a connecting muscular bundle crossing the plane of atrioventricular insulation [6]. The situation had been clouded, however, by the counterclaim of Kent that multiple myocardial pathways crossed the atrioventricular junctions [7]. Prior to these studies, furthermore, which were carried out in the last decade of the 19th century, it had generally been believed that atrioventricular conduction was neurogenic rather than myogenic, although the experiments of Gaskell had already provided evidence in favor of the myogenic concept [8]. It was the study of Tawara that clarified once and for all the morphology of the solitary myocardial pathway responsible for normal atrioventricular conduction. It is surprising, however, that Keith was unable to identify the conduction axis on the basis of the illustration provided by His (Figure 1A). 

Tawara studied multiple mammalian species and showed in greater detail how the pathway commenced in the atrioventricular node (Figure 1B), which is located in the base of the atrial septum. It then extended through the insulating place, and branched on the crest of the muscular ventricular septum. The branches were insulated by fibrous sheaths, which surrounded the fascicles before they ramified at the ventricular apices as the so-called “Purkinje” network [1]. It is surprising, given the excellence of these initial descriptions, that uncertainty continues to exist regarding the precise correlation between the anatomy and function of the atrioventricular node. We still do not know, for example, whether there are dual or multiple anatomical pathways through the node. If there are dual pathways, we have yet to identify the precise location and substrates for the so-called “fast” and “slow” options. With regard to development, the studies of Wessels and colleagues showed that the core of the node was derived from a ring of histologically and immunocytochemically specialized cells surrounding the embryonic interventricular foramen [9]. The significance of the derivatives of other parts of this ring, such as the so-called “retroaortic node”, has still to be established [10]. In particular, we remain uncertain regarding the mode of development of the atrial connections of the node. The potential influence of the vestibular spine, described by the father of the discoverer of the atrioventricular bundle [11] remains unexplored. Evidence is also now emerging of the evolutionary origin of the atrioventricular bundle in reptiles and other ectothermic vertebrates [12]. Taking advantage of our experiences in these various fields, therefore, we review here the anatomy, development, and evolution of the atrioventricular conduction axis.

## 2. The Location and Architecture of the Normal Atrioventricular Conduction Axis

### 2.1. The Structure of the Normal Atrioventricular Junctions

**Figure 2:**
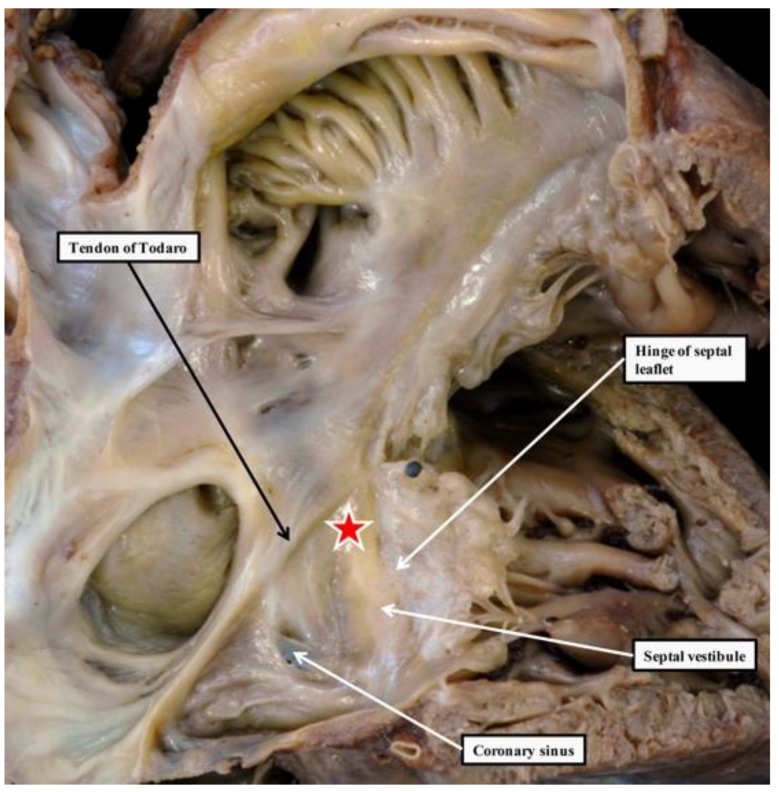

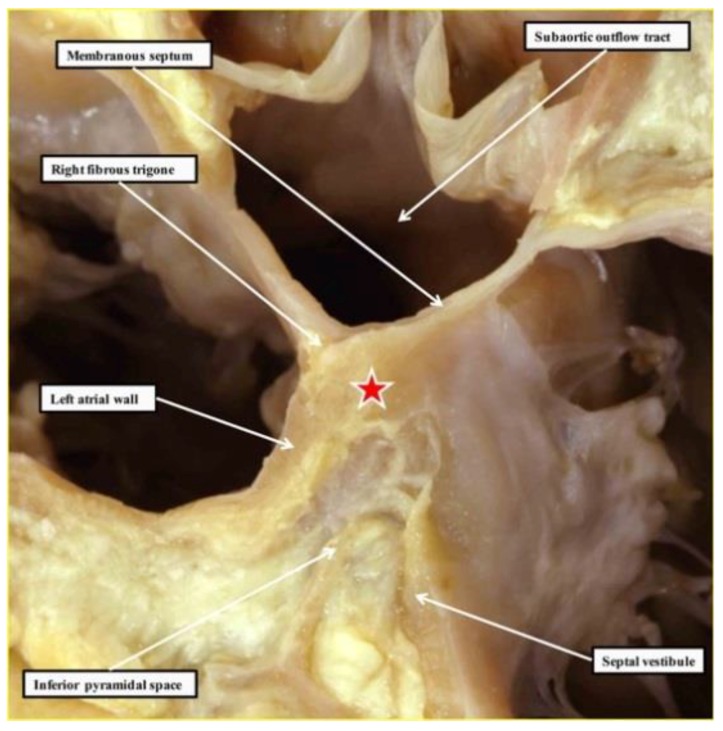
The top panel shows the opened right atrium, revealing the landmarks of the triangle of Koch, namely the tendon of Todaro and the hinge of the septal leaflet of the tricuspid valve. The atrioventricular node (red star with white borders), located at the apex of the triangle, is more-or-less centrally positioned within the cardiac base as viewed from the internal aspect of the right-sided chambers. As is shown in the bottom panel, which is a dissection made by removing the floor of the triangle, the node lies directly adjacent to a cranial continuation of the inferior atrioventricular groove. This fibroadipose area forms the inferior pyramidal space. The cranial extent of the space is bounded by the atrioventricular component of the membranous septum. It is this area that is penetrated by the bundle of His.

As already shown (Figure 1), Tawara had provided an excellent guide to the anatomical location of the axis [1]. His had demonstrated the location of its penetrating component, now justifiably usually described as the bundle of His [6]. Fully to appreciate the location of these various components of the axis in the human heart, it is also necessary to understand the structure of the atrioventricular junctions. Of particular importance is their relationship to the aortic root. The atrioventricular node, located at the apex of the triangle of Koch, is centrally located within the cardiac base. Despite this central location, the node itself is directly related to the area of fibroadipose tissue interposing between the septal component of the right atrial vestibule and the crest of the muscular ventricular septum. This is because a cranial continuation of the inferior atrioventricular groove extends to the inferior aspect of the central fibrous body. The central fibrous body itself is made up of the rightward extent of the area of fibrous continuity between the leaflets of the aortic and mitral valves in the roof of the left ventricle, an area usually described as the right fibrous trigone, and the membranous septum. The membranous septum itself forms the rightward margin of the subaortic outflow tract. The atrioventricular component of the membranous septum, furthermore, is the only atrioventricular septal structure to be found in the human heart. This is because the floor of the triangle of Koch is a sandwich rather than a true muscular septum (Figure 2).

### 2.2. Location of the Atrioventricular Node

**Figure 3:**
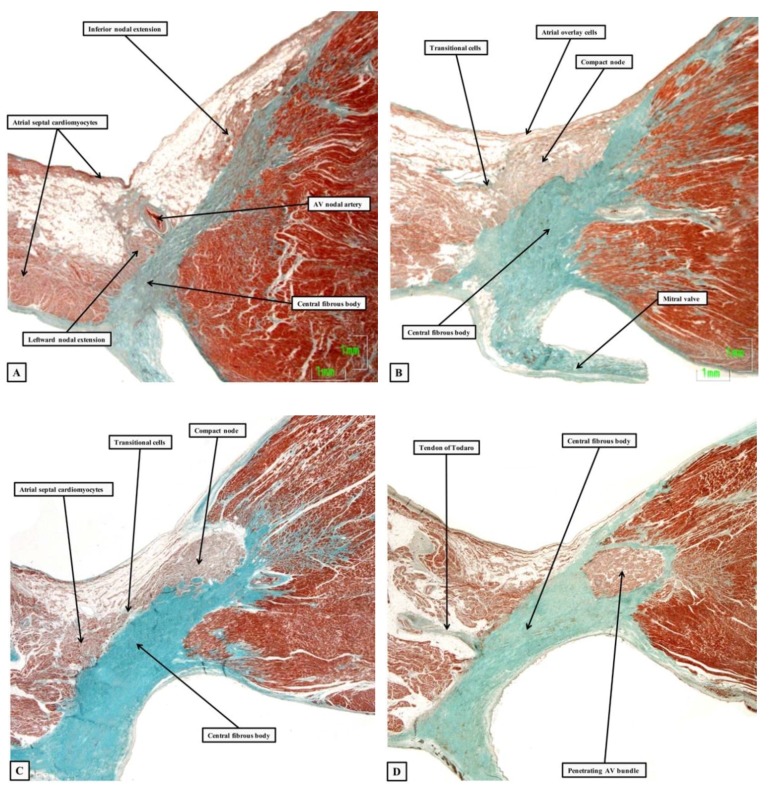
The sections are from a series prepared from a human heart, showing the atrial components of the conduction axis, and its transition to the bundle of His. They are orientated in attitudinally appropriate fashion, but with the chambers of the right heart shown to the top of the images. Panel (**A**) shows the rightward and leftward inferior extensions from the compact node, which is seen in Panel (**B**). Panels (**C**,**D**) show how the axis becomes the penetrating bundle once it is insulated by the fibrous tissue of the central fibrous body from the atrial cardiomyocytes.

The anatomical arrangement is such that the base of the atrioventricular node lies directly adjacent to the tissue plane interposed between the right atrial septal vestibule and the crest of the muscular ventricular septum. The surface of the ventricular septum itself is sloping, such that the body of the node forms a compact half-circle set with its base against the septal crest (Figure 3B).

The superficial atrial cardiomyocytes form overlaying fascicles that extend down from the atrial septum and insert into the hinge of the septal leaflet of the tricuspid valve. The cardiomyocytes of the atrial septum themselves form short transitional zones to the leftward half of the nodal half-oval. When the compact half oval itself is traced inferiorly, the columns of nodal cells branch to form short tracts that extend, on the left side, towards the hinge of the mural leaflet of the mitral valve. On the right side, they enter the right atrial septal vestibule, forming the inferior nodal extension (Figure 3A). When traced cranially, the cells of the compact node bunch together. Having collected together, they enter the substance of the central fibrous body, where the axis becomes the penetrating atrioventricular bundle (compare Figure 3C,D). The point at which the axis becomes insulated from the atrial cardiomyocytes is the best anatomical marker of the transition from the compact node to the penetrating atrioventricular bundle, or the bundle of His [1].

### 2.3. Penetration and Branching of the Conduction Axis

**Figure 4:**
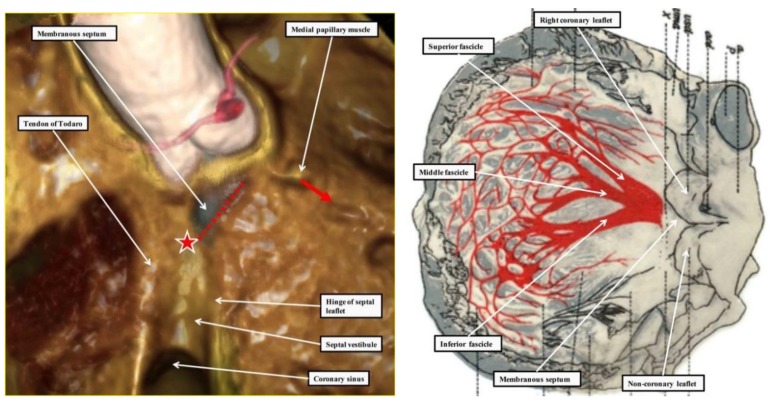
The left-hand image, prepared from a computed tomographic dataset obtained from an individual undergoing investigation for coronary arterial disease, shows how the location of the atrioventricular conduction axis can be predicted based on knowledge of the landmarks of the right atrial and right ventricular septal surfaces. The star shows the location of the atrioventricular node at the apex of the triangle of Koch. The red dotted line shows the location of the non-branching and branching components of the axis, which are carried on the crest of the muscular ventricular septum. The red arrow shows the site of emergence of the right bundle branch, marked by the medial papillary muscle of the tricuspid valve. The right-hand image is from the original monograph of Tawara [1]. It has been scanned and rotated through 90 degrees relative to the original, placing the image in more attitudinally appropriate orientation. The reconstructions made by Tawara show the origin and distribution of the left bundle branch within the left ventricle.

The atrioventricular conduction axis penetrates from the apex of the triangle of Koch through the atrioventricular component of the membranous septum. Having penetrated, the axis is positioned on the crest of the muscular ventricular septum. It has a non-branching component, which runs for a short distance along the septal crest before beginning to give rise to the fascicles of the left bundle branch. As was shown with exquisite accuracy by the reconstructions provided by Tawara [1], the fascicles of the left bundle branch, which are insulated by a fibrous sheath from the underlying septal musculature, cascade in a trifascicular fashion down the smooth left ventricular septal surface (Figure 4—right-hand panel). The most superior fascicle extends towards the supero-lateral papillary muscle of the mitral valve, while the inferior fascicle runs towards the infero-septal muscle. The middle fascicle descends between the two, forming a mesh-like network on the septal surface. The right bundle branch takes off from the bundle at the level of origin of the superior fascicle of the left bundle branch, passing through the substance of the ventricular septum to emerge beneath the medial papillary muscle of the tricuspid valve (Figure 4—left-hand panel). The axis itself, however, continues beyond the origin of the right bundle branch, extending into the subaortic outflow tract on the septal crest as the so-called “dead-end tract”. The extent of the terminal ramifications of the ventricular conduction system varies substantially between species, such that, for instance, the papillary muscles of the bovine heart is rich in Purkinje cells, whereas in other mammal hearts the ventricular myocardium harbors few Purkinje cells, or the Purkinje cells are poorly differentiated [13,14].

**Figure 5:**
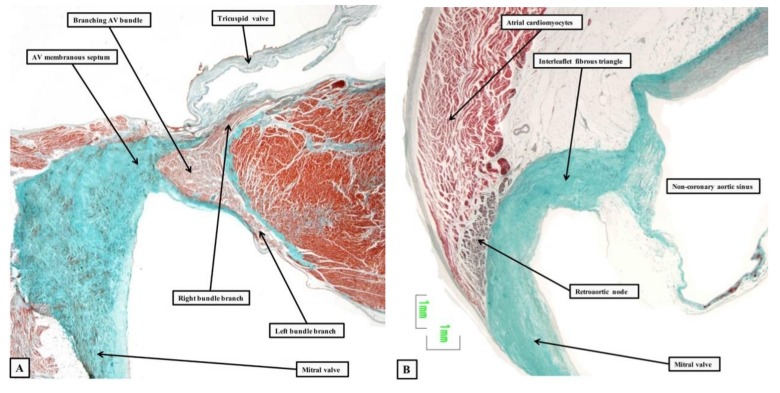
The images are from the same series as shown in Figure 4. Panel (**A**) shows the cranial continuation of the conduction axis, which has divided into the right and left bundle branches of the crest of the muscular ventricular septum. Panel (**B**) shows a section through the vestibule of the left atrium directly adjacent to the non-coronary sinus of the aortic root. The retroaortic node is seen on the atrial aspect of the insulating plane.

The bundle branches themselves continue to be insulated by fibrous sheaths from the substance of the muscular ventricular septum (Figure 5—left-hand panel).

The site of branching is usually directly astride the crest of the muscular septum. As already discussed, the left bundle branch fans out in trifascicular fashion, as demonstrated in the original monograph of Tawara (Figure 4—right-hand panel). The right bundle branch passes through the substance of the ventricular septum, emerging beneath the medial papillary muscle. It then extends through the substance of the septum as a small muscular strand before branching at the ventricular apex, usually with a prominent branch passing within the moderator band to the parietal ventricular wall. In a minority of cases, the central component of the axis branches on the left ventricular aspect of the septum, away from the septal crest [15]. The axis itself, nonetheless, continues beyond the branching component, extending into the aortic root as the so-called “dead-end tract” [16]. This component is part of the initial ring of primary myocardium that surrounded the embryonic interventricular communication. It is the remnant of this ring, lying with the vestibule of the right atrium, that were mistakenly taken by Kent to represent multiple pathways for atrioventricular conduction in the normal heart [7]. The remnants do exist, but are sequestrated within the vestibular myocardium on the atrial aspect of the insulating plane in individuals with normal atrioventricular conduction [17]. It is these remnants that produce the anomalous atrioventricular nodes found in situations when the heart is congenitally malformed, such as congenitally-corrected transposition or straddling tricuspid valve [18,19].

### 2.4. The Retroaortic Node

A particularly prominent remnant of the ring is to be found in the atrial myocardium at the junction of the vestibules of the tricuspid and mitral valves. This is the retroaortic node (Figure 5—right-hand panel), which has recently been implicated as a possible substrate for arrhythmias ablated from the non-coronary sinus of the aortic valve [20]. As yet, we do not have sufficient information to offer precise details regarding the size and full significance of this structure. Our current knowledge of cardiac development, however, now permits us to provide a detailed account of the formation of these various parts of the conduction axis. 

## 3. Development of the Cardiac Chambers

In the embryo, the beating heart is initially a myocardial tube. In the chambered heart, as was noted by Gaskell, the developing chambers are separated by junctions themselves comprised of myocardium with a phenotype comparable to that of the initial tube [8]. Keith had observed that the crest is the earliest part of the ventricular septum to be formed [4]. The junctions between the chambers and the ventricular septal crest, however, constitute a miniscule part of the total cardiac mass. The disparity in mass between the junctions and chambers is a testimony to the manner of cardiogenesis [21,22]. 

Cardiac chambers form locally within the heart tube, by virtue of greater rates of growth, in a process now commonly referred to as ballooning [23,24,25]. Molecular assessments of proliferation have validated the greater growth rates of chambers [26,27,28,29,30]. The transcription factors that promote formation and growth of the chambers, such as GATA4, TBX5, and NKX2.5, are expressed throughout the embryonic heart tube [29,31,32,33]. The recognition of the junctions between chambers, and, consequently, formation of the distinct chambers themselves, is made possible by repression of transcription factors. Repressors like TBX2 and TBX3 are particularly important [21,34,35,36,37,38,39]. These insights were much facilitated by the detection of mRNA by non-radioactive means on tissue sections [40]. Developing chambers undergo a gestational increment in the speed of electrical propagation [41,42]. This is reflected by the presence of gap junctions with low resistance between the cardiomyocytes making up the walls of the chambers, such as CX40 and CX43. The cardiomyocytes making up the atrioventricular junctional myocardium, in contrast, express the high resistance CX45, and at relatively low densities [13,43]. 

When traced from the venous to the arterial pole, the alternation between chambers and junctions corresponds to alternation between fast and slow propagation of the electrical activity. An adult-like electrocardiogram with atrial and ventricular activation separated by a substantial atrioventricular delay can be recorded from the embryonic heart soon after the chambers have started to form [41,42,44,45,46]. In the initial stages, as the ventricles begin their maturation [47,48,49], activation is initially detected near the forming septum. In the fetal stages, the first epicardial activation is deep at the ventricular apexes, presumably at some terminal parts of the left and right bundle branches [47,48,49,50]. The ventricular septum nonetheless remains the earliest activated part of the ventricles [51,52,53]. The patterns of activation therefore suggest that a pathway of preferential conduction has already been established at the site of the future atrioventricular bundle. Later development of the atrioventricular conduction axis sees an incremental regulatory role of nervous tissues and, as recently shown, resident macrophages, but the basic properties of conduction still arise from the patterning of the myocardium [25,54].

## 4. The Development of the Atrioventricular Conduction Axis

**Figure 6:**
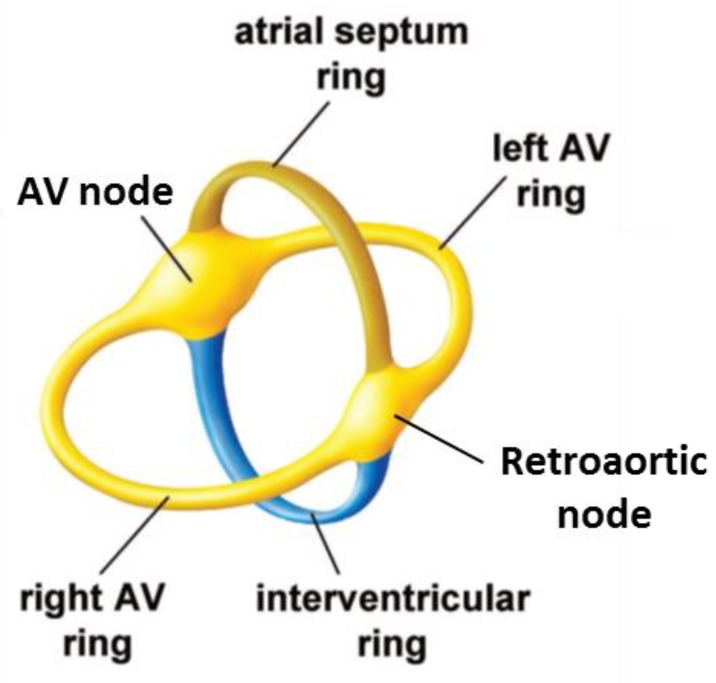
The domain of Tbx3-expressing myocardium partakes in dividing the heart in atrial and ventricular compartments, and also in the division of the atrial and ventricular compartment, respectively, by expression on the crests of the atrial and ventricular septum. Adapted from [58].

As the ventricular and atrial chambers begin to balloon out from the primary heart tube, the repressing transcription factors TBX2 and TBX3 start to be expressed in a circular domain between the chambers [25,55]. This area will become the atrioventricular canal [21,56]. The crest of the developing ventricular septum, formed between the ballooning cavities of the left and right ventricles, also expresses TBX3. This produces a domain that reaches the atrioventricular canal, which can be called the primary ring. The early electrical activation near the forming septum is likely due to preferential conduction along the primary ring [47,57]. The leading edge of the atrial septum also expresses TBX3 [21,56]. Collectively, the septal crests form a circular domain in a plane that is perpendicular to, and intersecting, the domain of the atrioventricular canal (Figure 6). The dorsal intersection of these rings marks the site of formation of the atrioventricular node. The ventral intersection will become the retroaortic node (Figure 6). 

**Figure 7:**
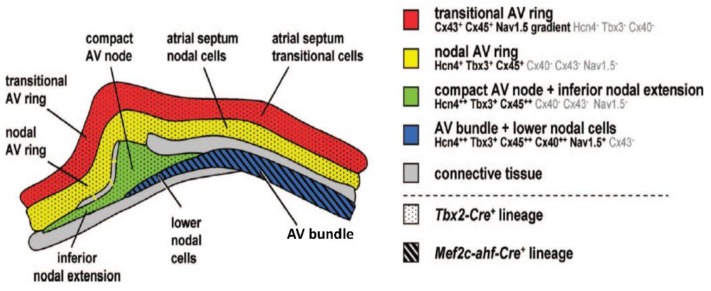
The identities of the myocardium of the atrioventricular junction of the formed murine heart. Adapted from [58].

Around the time of transition from the embryonic to the fetal stages of development, concomitant with growth of the muscular ventricular septum, TBX3 will start to be expressed on the left and right of the septal crest. This delineates the areas of formation of the proximal parts of the left and right bundle branches [21,56]. Later stages see the development of an insulating plane of fibro-fatty tissue around the atrioventricular junctions. This disrupts the myocardial continuity between the atrial and ventricular components, save at the site of penetration of the axis; in other words, the His bundle [59]. As part of this process, the myocardium of the atrioventricular canal becomes confined to the atrial side as the atrial vestibules (Figure 7). Subsequent to the development of the insulating plane, the electrical impulse generated by the sinus node passes through the atrial myocardium. If running dorsally, it reaches the myocardium of the atrioventricular canal, and then the atrioventricular node and, finally, the His bundle. If the impulse takes this course, then much of atrioventricular delay comes from the relatively slow propagation through the atrioventricular canal myocardium [60,61]. This is the so-called “slow pathway”. An alternative route is provided, however, subsequent to the completion of atrial septation. This route extends ventral to the oval fossa, and corresponds with the so-called “fast pathway” into the atrioventricular node. The development of this pathway remains to be clarified. Once the electrical impulse reaches the His bundle, it can activate the crest of the ventricular septum directly, as in mice [52], or propagate along insulated bundle branches such that early activation is closer to the apex, as in humans [51].

## 5. Molecular Characterization of the Atrioventricular Conduction Axis

**Figure 8:**
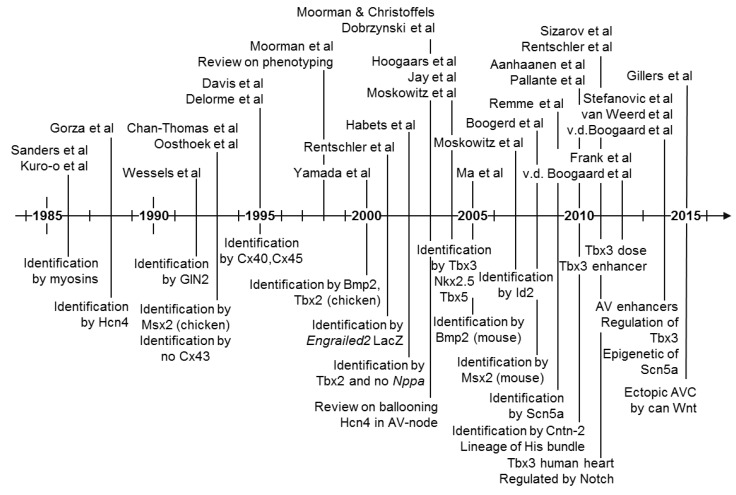
Chronology of key studies in the molecular identification of the atrioventricular conduction axis. Cited studies: [9,13,21,24,32,33,34,36,37,38,47,56,58,62,63,64,65,66,67,68,69,70,71,72,73,74,75,76,77,78,79]. There are recent and excellent reviews on the regulatory and transcriptional networks behind the formation of the cardiac conduction system [25,80].

The pale staining characteristics as seen in classic histological sections stained with hematoxylin-eosin or Masson’s trichrome reflect a low level of expression of cytoplasmic proteins, in particular proteins of the contractile apparatus. Such staining characteristics do not give gene or protein-specific information. Gene-specific information is typically obtained by quantitative PCR, which is dependent on skilled micro-dissection for crude spatial information. High-resolution spatial information requires histology or whole mount stains. This typically relies on in situ hybridization, or immunohistochemistry, for transcript- and protein-specific signals, respectively. Key studies in the molecular characterization and understanding of the atrioventricular conduction axis are shown in Figure 8. Identification of important factors has been made on multiple levels of organization. These include epigenetic regulation and differentiation by transcription factors such as *Bmp2*, *Tbx2*/*3*, *Tbx5*, *Nkx2.5*, *Notch*, *Wnt*, and identification of proteins, and their transcripts, which underscore physiological functions, such as the gap junctional proteins Cx30.2, Cx40, Cx43, Cx45, and the ion channels Hcn4 and Scn5a. 

## 6. Evolutionary Origin of the Atrioventricular Conduction Axis

The key components of the atrioventricular conduction axis are the atrioventricular node and the His bundle. They are distinct from each other, and the surrounding myocardium, on the basis of their anatomical, molecular, and electrophysiological characteristics [58,80]. The presence of each character can, in principle, be assessed in living vertebrates. By plotting the prevalence of each character on the phylogenetic tree of vertebrates, which itself is derived predominantly from fossil and genetic data, we can infer the most likely evolution of each character. As yet, however, there is no clear-cut synchrony in the appearance of these characteristics [12].

A cardinal feature of the atrioventricular node is the delay it establishes between atrial and ventricular contraction. All vertebrates have an atrioventricular delay, from hagfish to humans. A histologically discrete atrioventricular node, however, is present only in birds and mammals [24,81,82,83]. Irrespective of the presence or absence of an atrioventricular node, the atrioventricular delay is regulated by vagal stimulation to the point that an atrioventricular block can be induced [84,85]. Accordingly, Cranefield proposed that all vertebrates have the functional equivalent of an atrioventricular node [84]. We have recently shown that the atrioventricular delay of mammals and birds is some four times shorter, not longer, compared to reptiles. The latter animals are without an anatomically identifiable atrioventricular node (Boukens et al., in press). This permits the perhaps surprising conjecture that the evolution of the anatomically distinct atrioventricular node associates with a reduction, rather than an extension, of the atrioventricular delay. It is not yet known whether the difference in the duration of the delay is due to differences in expression, for instance, of gap junction proteins. 

The mammalian atrioventricular conduction axis is dorsal in the atrioventricular junction and it is the only route from the atrial myocardium to the ventricular myocardial mass. In turtle hearts, however, Mines showed that both ventral and dorsal parts of the atrioventricular junction can propagate the electrical impulse [86]. The mammalian atrioventricular junctions are different from the arrangements found in ectothermic vertebrates by virtue of the extensive disruption of the atrioventricular junctional myocardium by the plane of atrioventricular insulation [13,81,82,83,87,88]. The comparative analysis suggests that the development of the insulating plane was sufficient, during evolution, to produce a mammalian-like atrioventricular conduction axis. A possible negation of this conjecture comes from findings in crocodilians. Crocodiles, alligators, and the gharials, are the only vertebrates, together with mammals and birds, which have a full ventricular septum [89,90]. Crocodilians and birds are phylogenetically grouped in the archosaur clade. This is about 100 million years younger than the earliest branches of the mammalian lineage. The evolution of the full septum in archosaurs and mammals, therefore, is widely regarded as a case of convergent evolution [91].

The crocodilians are the only ectotherms with considerable development of a plane of atrioventricular insulation. In the American alligator, the ventral half of the atrioventricular junctional myocardium is interrupted by an insulating plane [92]. Accordingly, cuts to this myocardium do not affect the propagation of the atrioventricular impulse [12]. It had been noted over 100 years ago that the dorsal myocardium of the atrioventricular canal of crocodilians projected to the ventricular septum [93,94]. We now know that cuts to the dorsal atrioventricular junctional myocardium induce atrioventricular block [12]. The dorsal atrioventricular propagation occurs through a narrow subset of the atrioventricular junctional myocardium that can be considered to constitute a specialised pathway. This specialised pathway, however, is not insulated by connective tissues to form a bundle of His. Instead, it is found within a broad area of myocardial continuity between the atrial and ventricular walls in the dorsal part of the atrioventricular junction. The specialised pathway, nonetheless, can be identified by its expression of *Tbx3* [12], as is the case for the bundle of His of mammals and birds [21,95]. The *Tbx3-*defined bundle of the alligator heart, furthermore, is juxtaposed to the crux, which is the intersection of the atrioventricular groove and the dorsal interventricular coronary artery. This is again comparable with the bundle of His of mammals and birds [12]. Consistent with the presence of a specialised pathway in the alligator, the crest of the ventricular septum shows the earliest activation [12,50]. Early electrical activation also occurs deep in the ventricles in crocodilians, as in mammals and birds. This is in contrast to other reptiles, where early ventricular activation is in the vicinity of the atrioventricular canal [12,96,97]. The identification of the *Tbx3-*defined bundle corroborates some of the early anatomical works on crocodilians [93,94]. Other anatomical studies claimed to have found an atrioventricular conduction axis in non-crocodilian reptiles, but Prakash [98], for example, mistook an atrioventricular valve for the His bundle. The majority view is that non-crocodilian reptiles are without a specialised atrioventricular conduction axis [12]. Accordingly, the presence of *Tbx2*/*Tbx3* does not reveal a slender pathway in non-crocodilian ectotherms [87,99]. Findings in the crocodilians, therefore, offer an alternative view on the evolution and development of the atrioventricular conduction axis, namely that the atrioventricular conduction axis can evolve without being completely insulated by fibro-fatty tissues. Its formation may depend on little more than the presence of a full ventricular septum.

## 7. Conclusions

Generations of scientists have built an ever-larger body of knowledge on the foundational observations on the atrioventricular conduction axis by Wilhelm His Jr. and Sunao Tawara. Much of the anatomy of the atrioventricular conduction axis is well understood. Our knowledge is now being deepened by significant advances in electrophysiology and in particular molecular biology. These advances include major contributions from Antoon F.M. Moorman, who sought the complementary insights of anatomy, development, evolution, electrophysiology, and molecular biology [100]. The branch of knowledge generated by Wilhelm His Jr. and Sunao Tawara keeps on sprouting and evolving! It still leaves areas requiring clarification, most notably the anatomic substrates for the slow, as opposed to the fast, pathways into the atrioventricular node.
